# A Culture-Adapted Strain of *Babesia bovis* Has Reduced Subpopulation Complexity and Is Unable to Complete Its Natural Life Cycle in Ticks

**DOI:** 10.3389/fcimb.2022.827347

**Published:** 2022-02-10

**Authors:** Heba F. Alzan, Reginaldo G. Bastos, Jacob M. Laughery, Glen A. Scoles, Massaro W. Ueti, Wendell C. Johnson, Carlos E. Suarez

**Affiliations:** ^1^Department of Veterinary Microbiology and Pathology, College of Veterinary Medicine, Washington State University, Pullman, WA, United States; ^2^Parasitology and Animal Diseases Department, National Research Center, Giza, Egypt; ^3^Tick and Tick-Borne Disease Research Unit, National Research Center, Giza, Egypt; ^4^Invasive Insect Biocontrol and Behavior Laboratory, Agricultural Research Service, Beltsville, MD, United States; ^5^Animal Disease Research Unit, United States Department of Agricultural - Agricultural Research Service, Pullman, WA, United States

**Keywords:** *Babesia bovis*, *in vitro* culture, tick, attenuation, sexual stages, transmission

## Abstract

*Babesia bovis* natural field strains are composed of several geno-phenotypically distinct subpopulations. This feature, together with possible epigenetic modifications, may facilitate adaptation to variable environmental conditions. In this study we compare geno-phenotypical features among long-term (more than 12 years) (LTCP) and short-term cultured *B. bovis* parasites (STCP) derived from the *B. bovis* S74-T3Bo strain. LTCPs intraerythrocytic forms are smaller in size than STCPs and have faster *in vitro* growth rate. In contrast to its parental strain, the LTCP lack expression of the sexual stage specific 6cysA and 6cysB proteins and are unable to develop sexual forms upon *in vitro* sexual stage induction. Consistently, in contrast to its parental strain, LTCPs have reduced virulence and are not transmissible to cattle by vector competent *Rhipicephalus microplus* (*R. microplus*). Similar to previous comparisons among attenuated and virulent *B. bovis* strains, the LTCP line has decreased genomic diversity compared to the STCP line. Thus, LTCP may contribute to our understanding of adaptive mechanisms used by the parasites in response to environmental changes, protective immunity, virulence, and transmission by ticks. In addition, LTCPs may be considered as candidates for a non-tick transmissible vaccine against bovine babesiosis.

## Introduction

The tick borne apicomplexan *Babesia bovis* (*B. bovis*) is responsible for acute and persistent disease among cattle in tropical and subtropical areas and improved control is urgently needed. The complex life cycle of the parasite includes asexual reproduction while residing inside the erythrocytes of its bovine hosts, and sexual reproduction, occurring in the gut of the definitive *Rhipicephalus* tick vectors. In addition, the parasite is capable of morphing into other distinct stages, such as gametes, kinetes and sporozoites while developing in the tick environment, where it invades different tick tissues. This includes invasion of the ovary and egg tissues of the tick progeny, which are necessary steps leading to transovarial transmission of the parasite. While in its bovine vertebrate host, the parasite invades erythrocytes where it evades immune responses of the hosts, causing acute and persistent infections. This impressive range of parasite forms and adaptabilities are achieved with a relatively small ~8.3 MB genome. Interestingly, some of the parasite adaptive responses to selective pressures encountered during their life cycle use strategies based on genetic, phenotypic, antigenic, and strain population diversity. Such strategies are reflected in the ability of individual *B. bovis* parasites to express VES1/2 antigenic variants in order to escape the immune system pressure of the vertebrate host (Allred et al., 2000; Al-Khedery and Allred, 2006). Furthermore, diversity is also displayed as the existence of parasite strains containing a mix of genetically and phenotypically distinct subpopulations (Timms et al., 1990; Mazuz et al., 2012; Combrink et al., 2015; Mans et al., 2019). Likewise, this diverse strain composition may facilitate the selection of more apt parasite subpopulations as the parasites encounter distinct environments such as those in tick and vertebrate hosts, or *in vitro* cultures. In addition, phenotypic characteristics can be modified by different mechanisms, including environmentally driven epigenetic changes (Timms et al., 1990; Dalrymple et al., 1992). However, *B. bovis* strain compositions and characteristics remain poorly investigated. Clearly, better understanding of *B. bovis* strain composition and the ability of the parasite to adapt to changing environments are key to design improved methods of control.

The *in vitro* culture techniques based on the continuous microaerophilous stationary phase culture systems (Levy and Ristic, 1980) were previously established as novel and potent tools which allowed for a better understanding of the biology of *Babesia* parasites and estimating the parasite vulnerability to babesiacidal compounds (Silva et al., 2018; Batiha et al., 2019). The *in vitro* culture systems were also instrumental to accelerate discoveries leading to the development of gene modification systems (Suarez and McElwain, 2009; Hakimi et al., 2019), genomic (Brayton et al., 2007) and transcriptomic analysis (Pedroni et al., 2013), identification of novel vaccine candidate antigens (Alzan et al., 2019; Alzan et al., 2021) and overall, improved vaccines. However, *in vitro* cultures represent an artificial and relatively stable environment, in contrast to the dynamic and ample environments that the parasites find during their natural cycle in bovines and ticks, where parasites encounter distinct bottlenecks. Importantly, and in contrast to parasites found in circulation in infected animals, *in vitro* grown parasites develop in the absence of pressure of the immune system, and thus do not need to resort to activating mechanisms involved in antigenic variation, which are required to evade the immune system responses of their natural hosts. These mechanisms are known to require epigenetic modifications, including chromatin re-arrangements (Mack et al., 2020). Altogether, this suggests that *Babesia* parasites have a great ability to adapt in order to survive in the face of important selective environmental forces (Lau et al., 2011). While it was shown that *B. bovis* strains are highly diverse (Lau et al., 2011; Mazuz et al., 2012; Mans et al., 2019) in nature, limited work has been done regarding phenotypic and genotypic changes that may occur in “Long Term Culture Parasites” (LTCPs) compared to “Short Term Cultured Parasites (STCPs), and how the parasite biological mechanisms will be altered during adaptation to a different non-natural environment (Dalrymple et al., 1992).

In this study we focused on pheno and genotypical comparison between parasites that were freshly recovered from infected animals and cultured in the *in vitro* blood culture system for a short time (STC) with parasites of the same strain that were adapted for long term *in vitro* blood culture (LTC). The results of the study provide an example of how the parasite exploits its diversity, especially in terms of strain composition, resulting in dramatic changes in its virulence, morphology, and transmission phenotypes as a consequence of its adaptation to an altered environment.

## Materials and Methods

### Parasites

*B. bovis* S74-T3Bo long term culture parasites [LTCPs] strain is an isolate originally derived from a field animal in Texas and has been continuously maintained *in vitro* in the lab for almost 12 years, while the short term parasite strain [STCPs] were recovered from infected bovines inoculated with an original *B. bovis* S74-T3Bo stabilate (Goff et al., 1998). The Eko clonal line (Alzan et al., 2017) and the Mo7 biological clonal strain of *B. bovis* (Rodriguez et al., 1983; Hines et al., 1989) both were maintained as cryopreserved stabilates in liquid nitrogen (Palmer et al., 1982).

### Assessment of Morphological Characteristics

Thin smears and Giemsa-stained slides were prepared from both *B. bovis* STCP and LTCP then examined by light microscopy under 100x oil emersion lens for morphological evaluation. The length and the width average were calculated as pixels from the images taken by light microscopy. Statistical analysis was performed to estimate the differences between the two parasites groups using two tailed student t-test.

### *In Vitro* Growth Rate Comparison

The growth rates of *B. bovis* STCP and LTCP were compared by counting parasitemia of four *in vitro* replicates per strain every 24 hours over four days using stained blood smears and light microscopy. Cultures were maintained under standard conditions of temperature (37°C) and gas pressures (5% CO_2_) with a starting parasitemia of 0.5%. Statistical analysis was performed using t-test.

### Sexual Stage *In Vitro* Culture Induction for STCP and LTCP

To compare the ability of *B. bovis* LTCP and STCP to transform to sexual stages, *in vitro* sexual stage induction was performed. The STCP and LTCP were induced using Xanthurenic acid (Xa) as described previously (Mosqueda et al., 2004; Hussein et al., 2017; Alzan et al., 2021). Cultures were incubated at 26°C up to 24 h. and blood smears were taken from both parasite lines and stained with Giemsa to be visualized using light microscopy. Samples from STCP and LTCP induced and non-induced cultures were collected for protein extraction and western blot analysis by adding lysis buffer as described previously (Alzan et al., 2021). Similar STCP and LTCP induced and non-induced samples were kept for RNA extraction after adding Trizol to be used for RT-PCR analysis. All samples were stored at -20°C.

### Reverse Transcriptase PCR and Western Blot Analysis

To detect sexual stage specific transcript in *B. bovis* STCP and LTCP after induction, RT-PCR was performed to amplify 6cys *A* and *B* genes (Alzan et al., 2016). Total RNA was isolated from induced and non-induced *B. bovis* STCP and LTCP samples which were kept in Trizol at -20°C. The concentrations and the quality of RNA were determined using a Nano-drop spectrophotometer. Extracted RNA was treated with RNase inhibitor (Roche) and RNase-free DNase (Turbo DNA-free from Ambion) for 30 min at 37°C. The cDNA was synthesized using the SuperScript II RNase H reverse transcriptase (RT) first strand synthesis system (Invitrogen), using random hexamer primers according to the manufacturer’s protocol. Gene-specific primers as described (Alzan et al., 2016). All amplicons were cloned and confirmed by sequencing.

Protein lysates were prepared from induced and non-induced *B. bovis* STCP and LTCP by centrifugation at 16,000 rcf for 5min and washing of frozen blood pellet, kept for analysis, three times in 1x PBS and discarding supernatant. Cell lysis buffer with proteinase inhibitor was added to the pellet and used in the immunoblot assay (Alzan et al., 2016; Hussein et al., 2017; Alzan et al., 2021). Antibodies used were BABB 75 monoclonal against *B. bovis* RAP-1 as a positive control (Suarez et al., 1993) and a rabbit polyclonal that reacts with *B. bovis* 6cys A (Alzan et al., 2016; Alzan et al., 2021).

### DNA Polymorphism Detection and Phylogenetic Analysis

Sequence analysis of the polymorphic gene BBOV_III010230 (Sondgeroth et al., 2014), a NFkB binding protein (Nuclear Factor kappa-light-chain-enhancer of activated B cells) (Ueti et al., 2021), was compared between LTCP and STCP. This gene is very AT rich ~ 60.84% [398As, 358 Ts, 233Cs and 253Gs], has relatively short coding sequences and was selected to detect potential changes in *B. bovis* parasite populations before and after passage through ticks (Sondgeroth et al., 2014). Moreover, two SNPs and a 142-nucleotide (nt) deletion were shown to differentiate *B. bovis* virulent and attenuated parasite strains where shorter translated products are found in the attenuated strain (Sondgeroth et al., 2014). The primers used for PCR amplification were 230-For : CTATAGAATATAGCACGAAATTAAAAGG-Forward and 230-Rev: GTGGATAAAGACATGGATTAG-Reverse. The amplified sequences from the two strains were cloned into TOPO-TA cloning vector. Forty colonies were selected from both STCP and LTCP strains and sent for sequencing using ACAACTATTATTTAACAATGTTGC-reverse primers. In addition to STCP and LTCP strains, other strains of *B. bovis* were included: *B. bovis* Mo7 (Rodriguez et al., 1983; Hines et al., 1989) and 6cys EKO-cln line (Alzan et al., 2017). BLASTN [https://blast.ncbi.nlm.nih.gov] was used to blast BBOV_III010230 against different *B. bovis* strains and the sequences were analyzed phylogenetically using GenomeNet (https://www.genome.jp/tools/ete/).

### *In Vivo* Comparison of the Virulence and Transmission Phenotypes Between LTCP and STCP

For the acquisition feeding experiment, two ~4 months old splenectomized calves (C-1457 and C-1458) were experimentally infested with one gram of *R. microplus* larvae and then inoculated with ~10^7^ parasites from a S74-T3Bo frozen stabilate of STCP (Goff et al., 1998) and LTCP, respectively. The stabilate inoculations were timed to synchronize the peak *B. bovis* parasitemia with adult tick feeding to ensure that the highest parasitemia occurred during the time of greatest blood intake by the feeding ticks (Alzan et al., 2016). Fever and packed cell volume (PCV) parameters were monitored from both splenectomized calves before and after infection with *B. bovis*. Samples of blood for DNA extraction and blood smears were taken daily from the animals before and after inoculation to test for the presence of *B. bovis* DNA by PCR targeting *rap-1* (Figueroa et al., 1993) and to analyze the parasitemia. Ticks were collected from both groups after repletion and dissected for midgut analysis to determine *B. bovis* infection. Eleven days after inoculation, engorged female ticks were collected and incubated in 24 well plates for egg collection and hemolymph sampling (Howell et al., 2007; Alzan et al., 2021). At day 8 after tick dropping, samples from tick hemolymph were tested for the presence of kinetes. Eggs samples were separately pooled from engorged female ticks from each animal and kept for larvae production and for the subsequent transmission experiment.

For the transmission experiment, larva from one gram of the pooled tick eggs derived from C-1457 and C-1458 were separately applied on two spleen intact calves C-1481and C-1482 to test transmission of STCP and LTCP, respectively. To further confirm transmission phenotype, larvae from 10 grams of eggs derived from LTCP were applied on a third calf (C-1475). Clinical signs for babesiosis were monitored from all animals including fever and PCV. To detect the presence of *B. bovis* parasites in the three animals, blood samples were collected daily and tested by PCR targeting the *rap-1* gene. Serum samples were also collected from all animals to detect antibodies by ELISA. ELISAs were used to detect *B. bovis* seroconversion in animals used for transmission experiments. Competitive ELISA (cELISA) using RAP-1 (Goff et al., 2003) antigen was performed on serum collected for 17 days from animals C-1481, C-1482 and C-1475. As a control, positive serum was used from animal C167 (Goff et al., 2003) undiluted and at 1:2, 1:4 and 1:8 dilutions. Negative serum was used from the kits provided by Veterinary Medical Research & Development company (VMRD), Pullman, WA, USA.

## Results

### Phenotypical Differences Among *B. bovis* STCP and LTCP Lines

**Figure 1:**
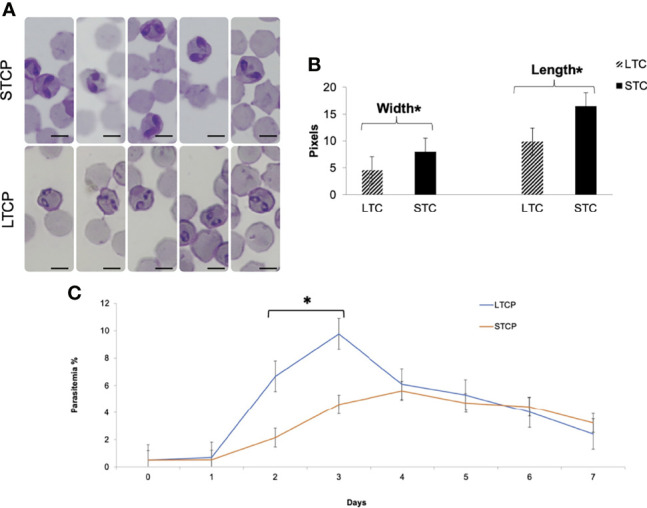
Phenotypic differences among *B. bovis* LTCP and STCP. **(A)** morphological differences of LTCP vs STCP detected by light microscopy. Bar: 5 µm. **(B)** The average length and width were calculated as pixels from the images taken by light microscopy and is represented in the “y” axis. (*) Indicates significant differences in average parasite length and width between LTCP and STCP. **(C)** *In vitro* growth curve comparison of LTCP and STCP *B. bovis* strains. (*) Indicates significant differences in the growth rate in the 2nd and 3rd day of the experiment.

Giemsa-stained *B. bovis* LTCP and STCP were compared by microscopy (**Figures 1A, B**). The parasites in the STCP line display a distinct size compared to the ones in the LTCP line (**Figure 1A**). The parasites of the STCP line were significantly larger than the ones in the LTCP line, in terms of width (P  lt; 0.05 (*t*_(48)_ = 10.83, *P*= 1.75^E-14^) and length (*t*_(48)_ = 15.34, *P*= 4.9^E-20^). Light microscopy images and size measurements of LTCP and STCP parasites are shown in **Figures 1A, B**, respectively.

The growth rates of the LTCP and STCP lines were also compared in *in vitro* cultures, under identical conditions (**Figure 1C**). The average differences of parasitemia between the LTCP and the STCP lines became significant (P < 0.05 (*t*_(14)_ = 4, P= 0.0002) on the 2^nd^ and 3^rd^ day of the experiment respectively (**Figure 1C**). Under the conditions used in this experiment the LTCP line had a significantly distinct, faster growth rate, in *in vitro* cultures, compared to the STCP line, suggesting an increased fitness in terms of parasite expansion.

Overall, the results demonstrate that the LTCP and STCP parasite lines have several phenotypical differences, including their morphologies and *in vitro* culture growth rate.

### The LTCP Lacks the Ability to Transition Into Sexual Forms Upon *In Vitro* Induction

**Figure 2:**
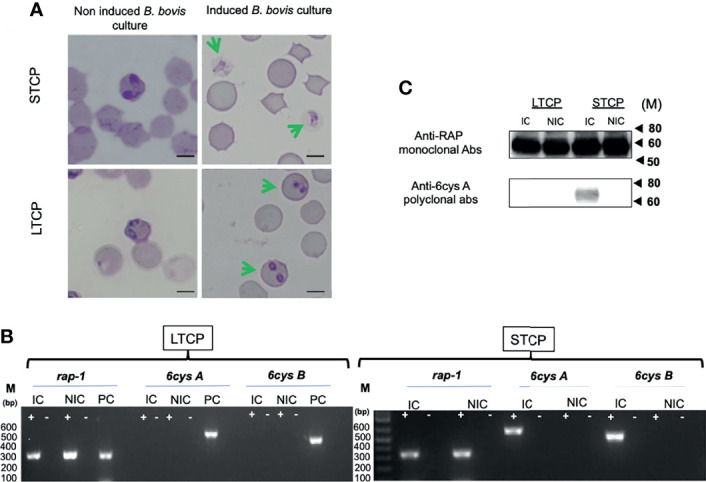
Comparative analysis of LTCP *vs* STCP ability to develop sexual forms in an *in vitro* sexual Xa-induction system. **(A)** Parasite morphologies detected by light microscopy upon *in vitro* Xa-induction of sexual forms. Non induced cultures were used as a control for both strains. Bar 5 µm. **(B)** Comparative RT-PCR analysis for the detection of transcripts of sexual stage maker genes, *6cys A and 6cys B* between LTCP and STCP. Transcripts derived from the *rap-1* gene were used as a positive control. RNA extracts were derived from (NIC) non-induced and (IC) induced *in vitro* cultures. M represents the sizes of molecular markers in base pairs. PC: Genomic positive control. **(C)** Western blot analysis for detection of 6cys A protein expression as a sexual stage marker using non-induced (NIC) and induced (IC) *in vitro* cultures of LTCP and STCP. The monoclonal antibody BABB75, reactive with *B*. *bovis* RAP-1, was used as a positive control. M represents the sizes of molecular markers in kiloDaltons (kD). The amplifications were performed without the addition of reverse transcriptase (-) and on cDNA generated from total RNA extracted from *B*. *bovis* infected RBCs (+).

The ability to produce sexual stages was compared between STCPs and LTCPs *in vitro* cultures when induced with Xa and grown at 27°C. The results are shown in **Figure 2**. Induction resulted in the production of typical sexual forms identical to the *in vitro* induced sexual forms described previously (Hussein et al., 2017) but only for the STCPs. In contrast LTCPs incubated with Xa did not produce such typical sexual-induced forms (**Figure 2A**), suggesting alterations in the mechanisms involved in sexual stage transitions for the LTCPs.

To further confirm and characterize this observation, we performed transcriptional analysis on the sexual stage marker genes 6cys *A* and *B* on total RNA extracted from Xa-induced STCP and LTCP. The RT-PCR analysis indicated the presence of transcripts of 6cys *A* and *B* (**Figures 2A, B**) only in the Xa-induced STCP, whereas no evidence of transcription of these two genes were found in RNA extracted from Xa-induced LTCP (**Figures 2A, B**).

Western blot analysis confirmed lack of expression of 6cys A in induced LTCP line parasites (**Figure 2C**), while expression of this sexual-stage specific protein in the STCP line was confirmed. Altogether, these experiments confirm the existence of important phenotypic differences between the LTCP and STCP lines.

### Differences in Parasite Subpopulation Composition Among the LTCP and STCP Lines

**Figure 3:**
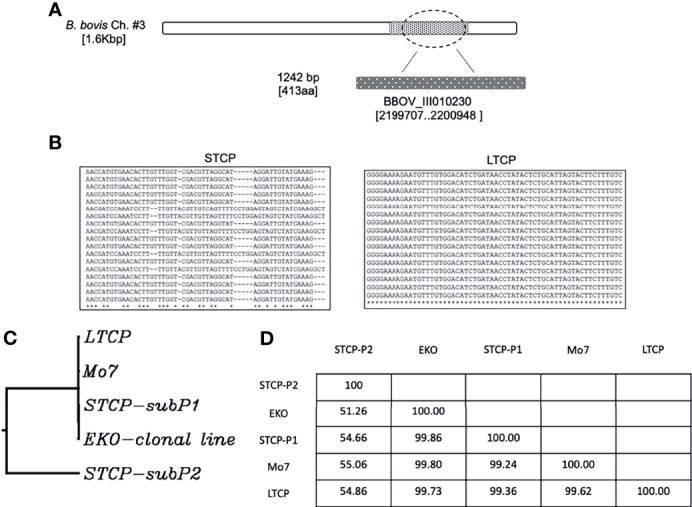
**(A)** Illustration of the location of the gene BBOV_III10230 used for the detection of DNA polymorphisms, gene diversity, and phylogenetic analysis among different *B. bovis* strains. **(B)** Nucleotide sequence representations of PCR amplicons derived from STCP and LTCP. **(C)** Phylogenetic comparison between the STCP (2 distinct genes amplified by PCR: subP1 and subP2), LTCP, MO7 and EKO clonal lines. **(D)** The Table shows identity percent comparison for the amplified genomic area among the different investigated parasite strains.

We compared the sequences of the marker gene BBOV_III010230 (Sondgeroth et al., 2014) among 40 individual clones derived from PCR amplicons from the STCPs and LTCPs strains. The experimental strategy and results of the comparisons are schematized in **Figure 3A**. Based on this genetic marker sequence, there were at least two variants detected in STCPs, in contrast, a single and distinct variant of this gene was found in LTCPs as demonstrated in the alignment (**Figure 3B**). A similar scenario, a single marker sequence detected for the BBOV_III010230 gene was also found in the two clonal lines, Mo7 and 6cys EKO. These findings support the notion that the STCPs for *B. bovis* parasites contain at least 2 genetically distinct parasite populations (termed subP1 and subP2) whereas no evidence of such distinct subpopulations was detected for the LTCPs. Interestingly, the LTCP contains the same BBOV_III010230 variants found in the attenuated clonal lines representing single parasite populations as shown in the phylogenetic analysis (**Figure 3C**). Also, phylogenetic analysis showed that the BBOV_III010230 gene of LTCPs, Mo7 and the 6cys Eko clonal lines group with the same subpopulation subP1, and in a separated clade than the subP2 variant, which was only found in STCPs. The identity percentages among the different strains are shown in table at **Figure 3D**. Altogether the data confirmed that the BBOV_III010230 gene in LTCPs, Mo7 and the 6cys Eko clonal line are homologous with the STCP-P1 (~99%), rather than the STCP-P2 sequence (with homology ranging 51 to 55%).

### Distinct *In Vivo* Virulence/Transmission Phenotypes Among LTCP vs. STCP Lines

**Figure 4:**
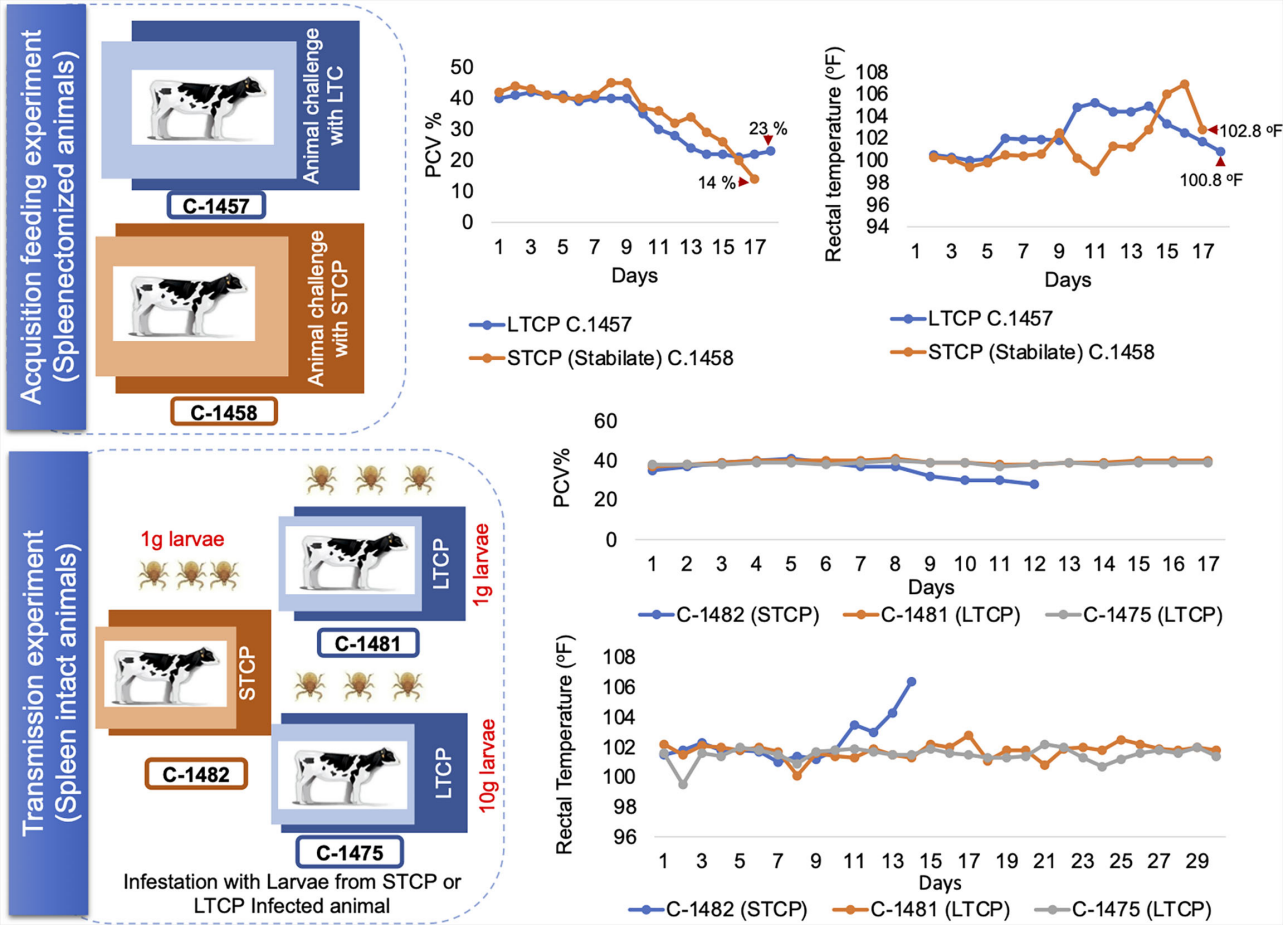
Schematic illustration and clinical parameters derived from the tick transmission experiments. The upper panel illustrate the acquisition feeding phase of the experiment, which was performed using splenectomized animals. Changes in the PCV and rectal temperature are illustrated on the right side of the panel. The lower panel illustrate the transmission phase of the experiment, performed on spleen intact animals. Changes in the PCV and rectal temperature are illustrated on the right side of the panel. Calf C-1482 infected with STC parasites *via* infected larvae was euthanized due to acute clinical signs at day 14 post application of control *B. bovis* infected larvae.

We first performed an acquisition feeding experiment by infecting two 4 months old splenectomized calves termed C-1457 and C-1458 with ~10^7^ parasites of the LTCP and STCP lines respectively. Both animals were previously infested with *R. microplus* larva 14 days before the inoculation of the parasites in order to synchronize adult tick acquisition feeding with *B. bovis* infection. Clinical signs of acute infection (rectal temperature and PCV) were compared daily in both animals (**Figure 4** - upper panel schematic representation).

The calf infected with the STCP had to be euthanized one day before the LTCP infected animal due to the development of severe acute clinical signs as shown in rectal temperature (102 °F) and PCV (14%) results (**Figure 4** upper panel two charts). The severe signs were synchronized with high levels of parasitemia. In contrast, infection with LTCP resulted in milder changes in PCV and temperature, suggesting that the LTCP had an attenuated virulence phenotype. PCR analysis to detect *B. bovis* DNA was performed on total DNA samples extracted from blood daily over 14 days for both experimentally infected animals. The results of this analysis are shown in **Figure S1A**. While no amplifications were detected in the samples at day 0 (before experimental inoculation), *B. bovis* DNA was amplified every day from day 2 until the end of the experiments for both animals.

Fully engorged adult female ticks started dropping from the infected animals at day 7 post inoculation and exposed 10 female ticks were dissected to extract tick midgut for further detection of *B. bovis* parasite. The DNA extracted from tick midgut sample were obtained daily during the three days of incubation period after female engorgement and were analyzed by PCR for both STCP and LTCP groups. The results of the PCR analysis for each of the 10 ticks used for DNA extractions at days 1, 2 and 3 of incubation are shown in **Figure S1B**. While the intensity of the signals obtained in the PCRs decreased on each day for both groups, the data suggests that both midguts derived from the ticks feeding on the bovines infected with either the LTCP (C-1457) or the STCP (C-1458) have parasite DNA until day 3 of incubation.

**Figure 5:**
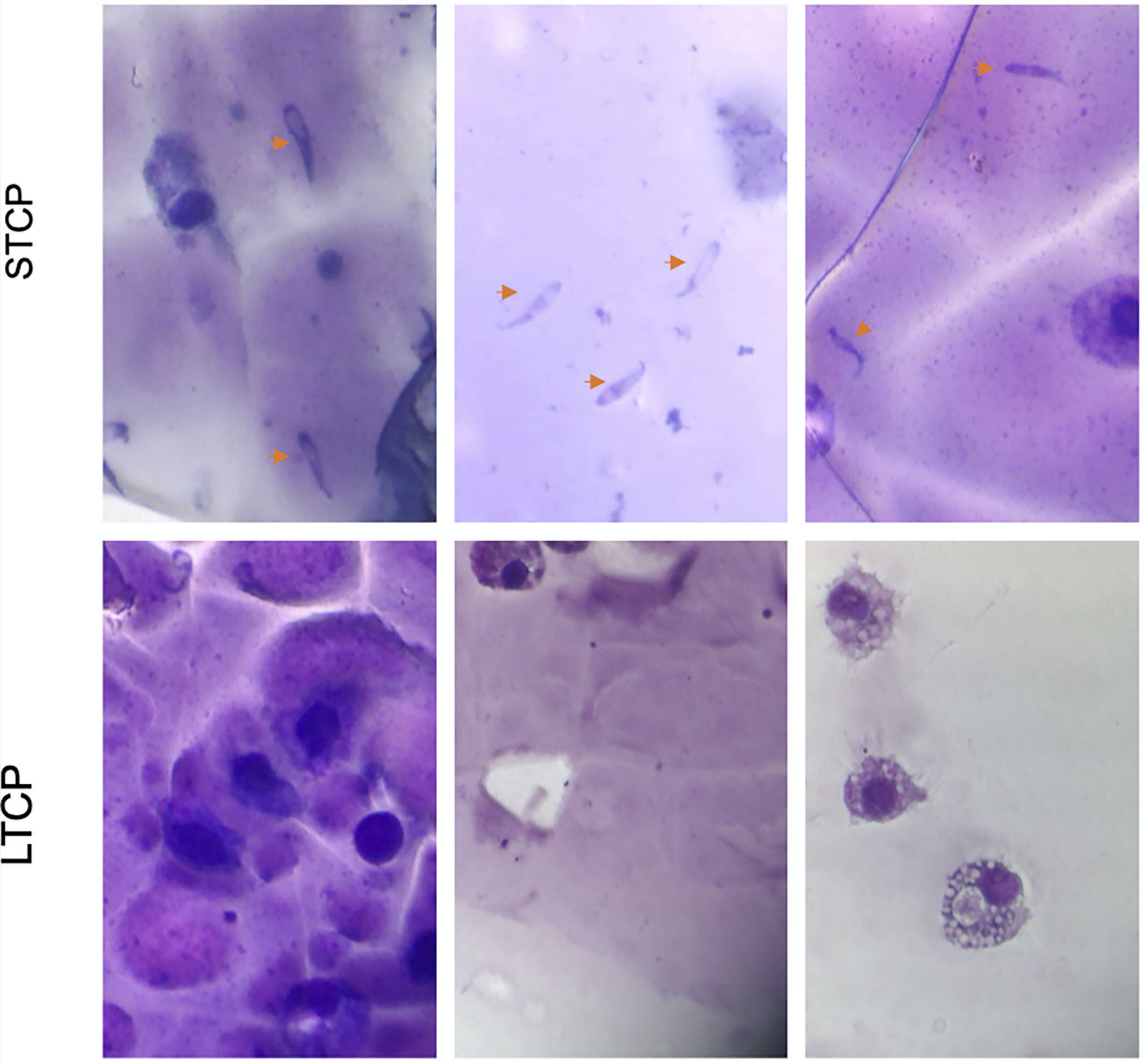
Analysis of animal and tick parameters for *B. bovis* infections in the tick transmission experiments. Giemsa-stained slides of a representative hemolymph sample to detect kinetes collected from ticks feeding on either C-1457 or C-1458 animals using 43x power objective of the light microscope. The upper panel represent samples collected from ticks fed on animal C-1458 infected with S74-T3Bo STCP stabilate. The lower panel represent samples collected from ticks fed on animal C-1457 infected with LTCP strain “lack of kinetes”.

The presence of kinete stages in the hemolymph were investigated in the ticks that fed on both STCP and LTCP infected calves. Hemolymph smears collected from the ticks were stained with Giemsa stain and analyzed by light microscopy (**Figure 5**). Slides from ticks that fed on the animal infected with STCP strain (C-1458) showed abundant kinetes in their hemolymph (upper panel) as expected, whereas hemolymph slides from tick samples collected from the animal infected with LTCP strain (C-1457) showed no kinetes at all in any examined slides (lower panel). Importantly, these results were consistent in all groups of ticks that were collected for 4 days (**Figure 5**). These results suggest that the LTCP lost its transmissibility phenotype despite maintaining the ability to infect a bovine host and be present in the guts of the ticks feeding on the experimentally infected bovine.

We then performed a transmission feeding experiment using larvae derived from both cattle infected with STCP and LTCP to infest three additional calves (**Figure 4** - lower panel schematic representation). Calf C-1482, that received larvae from 1g of eggs derived from ticks fed on the *B. bovis* STCP infected animal showed signs of acute babesiosis, including high rectal temperature, peaking at 106.4 °F at day 14 post infestation (**Figure 4** lower panel charts). It ultimately had to be euthanized early in the experiment due to high parasitemia as demonstrated by the PCR results (**Figure S2A**), and clinical signs compatible with acute babesiosis. In contrast, neither calf C-1481 that received 1g, nor calf C-1475 that received larvae from 10g of eggs derived from ticks that fed on the *B. bovis* LTCP infected animal showed any clinical signs of bovine babesiosis, and consistently displayed normal rectal temperatures with no changes in PCV, as shown in **Figure 4** lower panel chart and **Figure S2B**.

We further analyzed the occurrence of seroconversion as a sign of *B. bovis* infection in sera from the calves infested with larvae derived from LTCP and STCP infected calves, using ELISA. The results of the serological analysis are shown in **Figure S3**. Calf C-1482, which received 1g larvae coming from tick that fed on the *B. bovis* STCPs infected calf, suffered from severe acute babesiosis and had to be humanely euthanized. Consequently, serum samples from this calf were only collected for 10 days, before the development of detectable levels of antibodies by ELISA using RAP-1 antigen (**Figure S3**). In addition, neither calf C-1481 nor calf C1475, that received 1g and 10 g of larvae, respectively derived from ticks that fed on the *B. bovis* LTCP infected animal displayed clinical signs compatible with babesiosis, nor developed detectable level of antibodies by two distinct ELISA tests based on RAP-1 (**Figure S3**).

## Discussion

Taking together, the findings in this study suggest the occurrence of effective selection and adaptive mechanisms operating during the process of *in vitro* culturing of *B. bovis*, resulting in important phenotypic and genotypic changes among the LTCP and parental parasites, as represented by the STCP. In the case of *B. bovis*, long term parasites lost their transmission and virulence phenotypes. Consistent with these observations, previous work already demonstrated that virulent *B. bovis* and *B. bigemina* strains can become attenuated after being maintained in *in vitro* culture systems, but the causes and biology underlying these phenotypic changes remains poorly characterized (Kuttler et al., 1983; Yunker et al., 1987; Kuttler et al., 1988; Alarcon et al., 1996; Alarcon et al., 2003; Alvarez et al., 2004; Ojeda et al., 2010; Alvarez et al., 2020).

It is possible that the differences in morphology, transmission, and virulence phenotypes found among the parental and cultured strains may be due to mutations, epigenetic mechanisms operating in the parasites during the process of culturing, the selection of a subpopulation of parasites that were pre-existing in the parental strain, or a combination of these and other possible still unknown mechanisms (Dalrymple et al., 1992; Huang et al., 2013). It may be assumed that *in vitro* culture conditions provide a stable environment that somehow mimic natural conditions in the host, but in fact, *in vitro* growing parasites are not exposed to the pressures of immunological and/or physiological responses of the host and are unable to fulfil the entire life cycle of the parasite involving sexual reproduction in the tick vector and transmission.

An efficient process of cell invasion is crucial for the survival of the parasite inside their hosts. Initial studies on the *in vitro B. bovis* culture, Erp et al., (Huang et al., 2013) found that continuous parasite growth was maintained for 32 days and the PPE only reached to 15%. Consistent with our data, the parasite virulence in the cattle remained unchanged, likely because it was cultured for only a relatively short period of time. To our knowledge, no previous studies have compared the differences between LTCPs and their parental strains regarding their growth rates in *in vitro* culture. The data reported here shows that LTCPs have a significantly faster growth rate than the parental STCPs strain in *in vitro* cultures. This phenomenon was also reported in *B. duncani* developing in *in vitro* cultures (Abraham et al., 2018). The faster growth rate, with production of higher PPEs, may be due to the selection of a parasite subpopulation with more efficient erythrocyte invasion parasite subpopulation under the environmental conditions used in the *in vitro* culture system, and/or more efficient metabolism and asexual reproductive fitness for the culture-adapted parasite line, among other possible factors. Whether the LTCP are also more efficient for developing in the bovine host, which, in contrast, represent a natural environment, remains unknown, but their poor virulence compared to their parental uncultured strain, suggests that this might not be the case.

Another striking finding from this study is the inability of the *B. bovis* LTCP to produce gametocytes in an *in vitro* induction system, which was consistent with their loss of the transmission phenotype. Similar findings were previously reported for *B. divergens* (Jalovecka et al., 2016) and *Plasmodium falciparum* (Talman et al., 2004). The reduction or absence of the gametocytes upon induction was also observed in *P. falciparum* and *P. berghei* maintained in long term culture for several months to more than one year (Lobo and Kumar, 1998). Moreover, in contrast to the STCP, the *B. bovis* LTCP were not able to express the sexual stage marker genes *6cys A* and *B* (Alzan et al., 2016), as demonstrated by RT-PCR and Western blot analysis. These differences might be an important predictor, and perhaps a cause, for the inability of LTCPs to develop into gametocytes upon Xa-induction. The results in our study are consistent with previous findings by Stewart (Stewart, 1978), comparing the presence of *B. bovis* the tick gut between a virulent (T) and non-transmissible (NT) strain that was attenuated by repeated passage in the cattle. In our study we also detected *B. bovis* DNA in the guts of ticks feeding on infected cattle up to three days after replete female ticks had dropped. The study by Stewart (Stewart, 1978) showed that the NT vaccine strain parasites were plentiful in the tick gut throughout the period of the experiment in the tick gut, in contrast to the virulent T strain parasites, which decreased in numbers with time and were difficult to find. In addition, DNA of the T strain parasites was found in the hemolymph in contrast to the NT strain. Those results suggest that the continuous blood passaging of the NT strain in cattle during the process of attenuation resulted in selection of a parasite subpopulation that is incompetent for penetrating epithelial cells of the tick gut (Stewart, 1978). Our data is also consistent with this previous study showing that parasites that cannot fulfill the natural parasite cycle in the tick midgut, such as the LTCPs, remain trapped in the midgut, unable to pass to the hemolymph compartment. In contrast, parasites that are able to undergo sexual stage development, such as the STCPs, can pass through this bottleneck, form zygotes and penetrate the hemolymph compartment of the tick, then invade the ovaries, ultimately resulting in the transovarial transmission of the parasite.

Most, if not all, *B. bovis* strains in nature, including the wild type parental S74-T3Bo *B. bovis* strain used in our experiments, contain a mixture of parasite subpopulations representing several distinct genotypes (Mazuz et al., 2012). The *B. bovis* parasites undergo sexual reproduction resulting in genetic recombination inside the tick midgut. This recombination forms the basis for the continuous generation of novel field genotypes and maintains genetic diversity among *Babesia* parasites. The existence of dynamic and distinct vertebrate and invertebrate environments during *Babesia* life cycle, urge the parasites to have different strategies to survive the challenges of the immune systems of both hosts. Some of these strategies are based on generation of phenotypic and genotypic diversity. The reduction in complexity of the LTCP populations’ genome was suggested by sequencing of the polymorphic area located in BBOV_III010230 gene. Our results indicate that the LTCPs have a single variant of such sequence *vs* the at least two distinct sequence populations in STCPs. A similar, analysis of the BBOV_III010230 gene in clonal populations also results in the identification of a single variant, suggesting that the LTCPs might also have a clonal-like population composition. However, the aim of this experiment was not to map all the locations in LTCPs that lost polymorphism character, but to shed light on the possible changes taking place at genomic level using a short polymorphic region of the genome as a marker for the polymorphism. Also, and consistent with our current observations, loss of diversity among 18s ITS-2 polymorphic transcripts was also shown in a previous study using S2-T2bo cultured parasites, a predecessor of the S74-T3Bo strain, when compared to parasites derived from infected bovine, tick larvae and eggs (Laughery et al., 2009). Admittedly, the approach used in this study was limited, and confirmation of this conclusion will require a larger scale multilocus, or even or full genome sequence comparisons. The limited population repertoire, associated with loss of diversity, may be responsible for some of the phenotype changes, such as the lack of ability of these parasites to be transmitted by a competent tick vector, observed in the LTCP strain. This observation is consistent with the South African *B. bovis* S24 vaccine strain showing the presence of a single A558 Bv80 allele that comprises a subpopulation that displays low virulence and the non-transmissibility phenotype (Combrink et al., 2014; Mans et al., 2019). A single non-tick transmissible clone, (9626-S17.2-cl) of this South African vaccine strain was not able to infect the tick in two feeding attempts (Mans et al., 2019). Altogether these data suggest the preexistence of different parasite subpopulations, each with distinctive molecular properties and phenotypes. The selection imposed during the attenuation process the selective forces involved will likely favor the fittest pre-existing parasite populations, and these may include those parasites equipped with the genetic ability to adapt and respond quickly to the operating selective forces (Gill et al., 1987).

In contrast to the STCP, the data suggests that the LTCP lost the ability to express genes required for sexual stage development and to develop sexual forms in an *in vitro* sexual stage induction assay. No evidence of *B. bovis* transmission was detected using either one or ten grams of larvae derived from LTCP infected calf. It was reported previously that the South African S24 vaccine strain (with “24 passages in cattle”), was not transmissible by ticks (Mason et al., 1986; Combrink et al., 2015) but can be co-transmitted with the field strain. This process resulted in the emergence of a new transmitted population with a distinct genotype (Combrink et al., 2015). Similar observations were recorded for the Australian *B. bovis* vaccine strains (Ka strain), that was produced by rapid passaged for 20 –30 times in cattle and which contains both, transmissible and non-transmissible subpopulations (Gill et al., 1987; Timms et al., 1990). These two vaccine strains provide support for a hypothesis that transmissible subpopulations may work together with non-transmissible subpopulations by providing transmission factors, either exogenously, or *via* sexual recombination, however the mechanism remains unknown (Mans et al., 2019). The LTCP strains could be a reasonable candidate to confirm this observation when performing similar experiments in combination with transmissible field strains. During the development of these transmission experiments, we noticed that the LTCP line had reduced virulence compared to the STCP, but the experiments in the current study involved a single animal per strain tested. Such transmission experiments do not require large numbers of animals, since the variable under observation only relates to the number of ticks containing the parasite population. However, we previously observed diminished virulence in the LTCP line in unpublished experiments, a larger number of experimental animals will be required to confirm the observation of reduced virulence.

Attenuation of *Theileria* parasites has also been shown to occur by prolonged culture of infected cells *in vitro*. In addition, this is marked by a loss of complexity in the parasite population structure and alteration to infection associated host cell gene expression linked to pathogenesis (Echebli et al., 2014). However, a specific genotype or consistent change to gene expression that confers a loss of virulence has not been identified for attenuated *T. annulata* lines (Hall et al., 1999). Likewise, direct genomic and transcriptomic comparisons between virulent and attenuated strains in *B. bovis* suggested that virulence (or attenuation) mechanisms may not be shared among all populations of parasites at the gene level, but instead may reflect expansion or contraction of the population structure in response to shifting milieu (Lau et al., 2011). In conclusion, alteration in population structure and generation of attenuated lines during *in vitro* culture has been recorded for both *Babesia* and *Theileria* parasites and may point to a related process that results in the loss of parasite virulence.

In summary, we have demonstrated the transmission phenotypes of the LTCPs vs STPCs, and we have reported the loss in the ability to produce sexual stage parasites, leading to the inability for tick-born parasite transmission. It is interesting that the phenotypic changes found, such as changes in the *in vitro* growth rate, reduced virulence and transmission phenotype, are all suggestive of a parasite economy based on a “use it or lose it” strategy, since in theory none of these features provide advantages to parasites that are developing exclusively in *in vitro* cultures. This parasite strategy makes full sense, at least intuitively, and provides an interesting example of adaptation of a population to differential environments. It is feasible that long- term maintenance of *B. bovis* parasite populations in bovine RBC culture resulted in the selection for favorable mutations at genomic or metagenomic levels that were advantageous to the parasite during the growth in culture. Alternatively, there is the possibility that selection for a parasite subpopulation that is more efficient for development in *in vitro* culture conditions, unintentionally selects for a pre-existing attenuated and non-transmissible sub-population. However, the phenotypic differences may also be due to changes in the regulation of gene expression because of mutations in key regulatory regions or epigenetic modifications. More experiments, including full genome sequencing of the LTCP followed by further analysis on the parental strain will be required in order to sort out among these possibilities. Further studies are also needed to clarify whether epigenetic changes that affect the genome of the long-term cultured parasites might be related to the phenotypic differences among LTCP and STCP. These analyses may lead to the discovery of virulence factors and genes required for transmission, which may aid in the development of novel subunit vaccines or genetically manipulated parasites for defined non-transmissible live vaccines.

In conclusion, regardless that *in vitro* cultures have provided important contributions to our understanding of the biology and pathogenicity of *Babesia* parasites, it is clear that, the *in vitro* adapted parasites differ from the parental strain in terms of genetic composition and phenotype. Suggesting that researchers need to be cautious when using LTCP as surrogates of the parental strain when performing studies leading to the definition of virulence or transmission factors, as well as studies aiming at defining vaccine candidates using *in vitro* neutralization approaches. The LTCPs might represent an alternative way of producing attenuated and non-transmissible live vaccines. In addition, together with the parental STCPs strain used in this study, it can be used for performing comparative investigations using “omics” approaches. The LTCP could also become a potent tool to investigate immune mechanisms involved in the development of protective immunity against *B. bovis*. These future studies will likely contribute to our understanding of the biology and mechanisms of virulence, immunity, and transmission of *Babesia* parasites.

## Data Availability Statement

The datasets presented in this study can be found in online repositories. The names of the repository/repositories and accession number(s) can be found in the article/**Supplementary Material**.

## Ethics Statement

All animal experiments were approved by the Institutional Animal Care and Use Committee, University of Idaho (Protocol IACUC- #2013-66).

## Author Contributions

Conceptualization, HA and CS. Investigation, HA, CS, and GS. Formal analysis, HA. Visualization, HA, CS, and GS. Supervision, CS and GS. Funding acquisition, HA and CS. Writing, review and editing— HA, CS, JL, RB, WJ, and MU. All authors contributed to the article and approved the submitted version.

## Funding

This work was supported by ARS-USDA CRIS 2090-32000-039-000-D. RB is supported by the USDA National Institute of Food and Agriculture (NIFA) (Award Number: 2020-67015-31809; Proposal Number: 2019-05375, Accession Number: 1022541. We acknowledge financial support from the National Research Center and Ministry of High Education and Scientific Research of Egypt.

## Conflict of Interest

The authors declare that the research was conducted in the absence of any commercial or financial relationships that could be construed as a potential conflict of interest.

## Publisher’s Note

All claims expressed in this article are solely those of the authors and do not necessarily represent those of their affiliated organizations, or those of the publisher, the editors and the reviewers. Any product that may be evaluated in this article, or claim that may be made by its manufacturer, is not guaranteed or endorsed by the publisher.
