# Supplementary material for: A Culture-Adapted Strain of Babesia bovis Has Reduced Subpopulation Complexity and Is Unable to Complete Its Natural Life Cycle in Ticks
Source: Front Cell Infect Microbiol. 2022 Feb 10;12:827347. doi: 10.3389/fcimb.2022.827347 (PMC8867610; doi:10.3389/fcimb.2022.827347)
Supplement: Supplementary file 1 [file DataSheet_1.pdf]

Supplementary Material

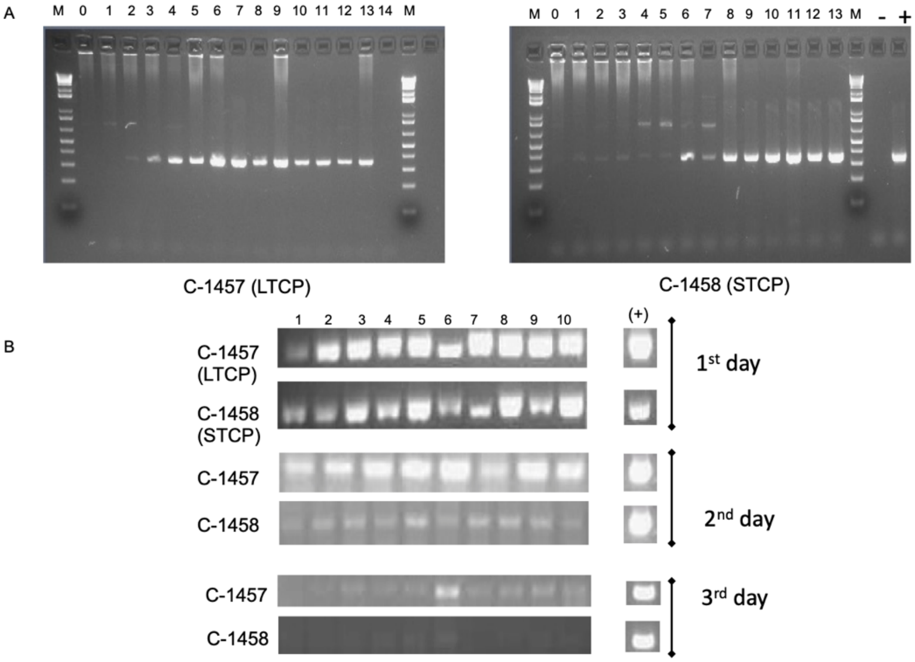

Supplementary Figure 1. A. PCR analysis of calves infected with *B. bovis* from the LTCP and STCP strains amplifying gDNA isolated from whole blood of calves C-1457 and C-1458 using *rap1* specific primers. The numbers on the top represent days after experimental infection. M represents the sizes of molecular markers in base pairs, (-) represents negative template reaction and (+) represents positive control using 5ng S74-T3Bo gDNA. B. Tick gut analysis from two tick groups that fed on animal infected with LTCPs or STPs. PCR amplifications using *rap1* specific primers (nested) with gDNA isolated from tick midgut from ticks fed on calves C-1457 or C-1458. The numbers on top indicate material used from a single dissected tick. First, second and third day after repletion is indicated on the right. Positive control (+) was performed using *B. bovis* S74-T3Bo gDNA obtained from cultured parasites.

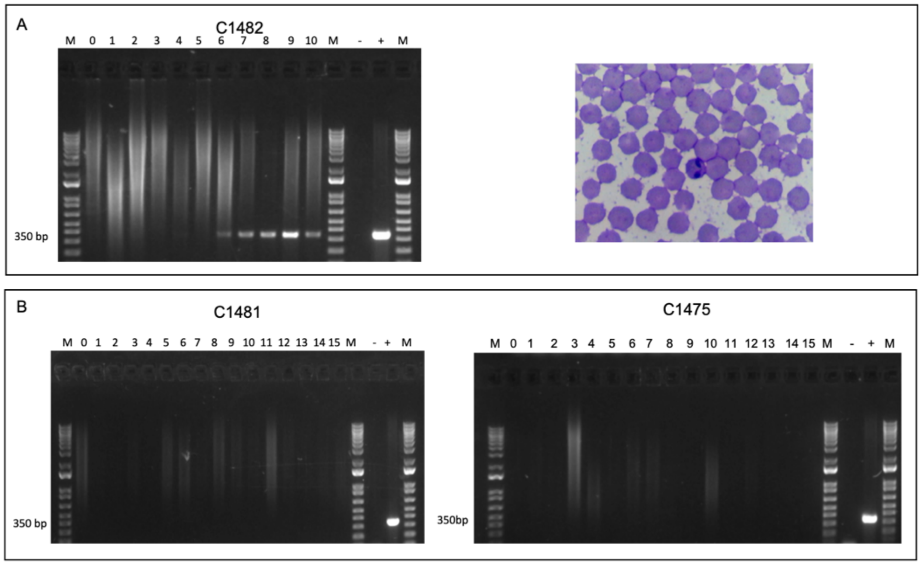

Figure S2. Analysis of gDNA isolated from whole blood of the calves used in *B. bovis* parasite transmission experiment by PCR using *rap1* specific primers (nested). A. PCR analysis of DNA extracted from calf C-1482 blood. The photo on the right indicates the presence of *B. bovis* parasitized erythrocytes in the blood of calf C-1482. B. PCR analysis of recipient calves C1481 (received one gram tick larvae) and C-1475 (received ten grams tick larvae). M represents the sizes of molecular markers in base pairs. Positive control (+) using *B. bovis* S74-T3Bo gDNA.

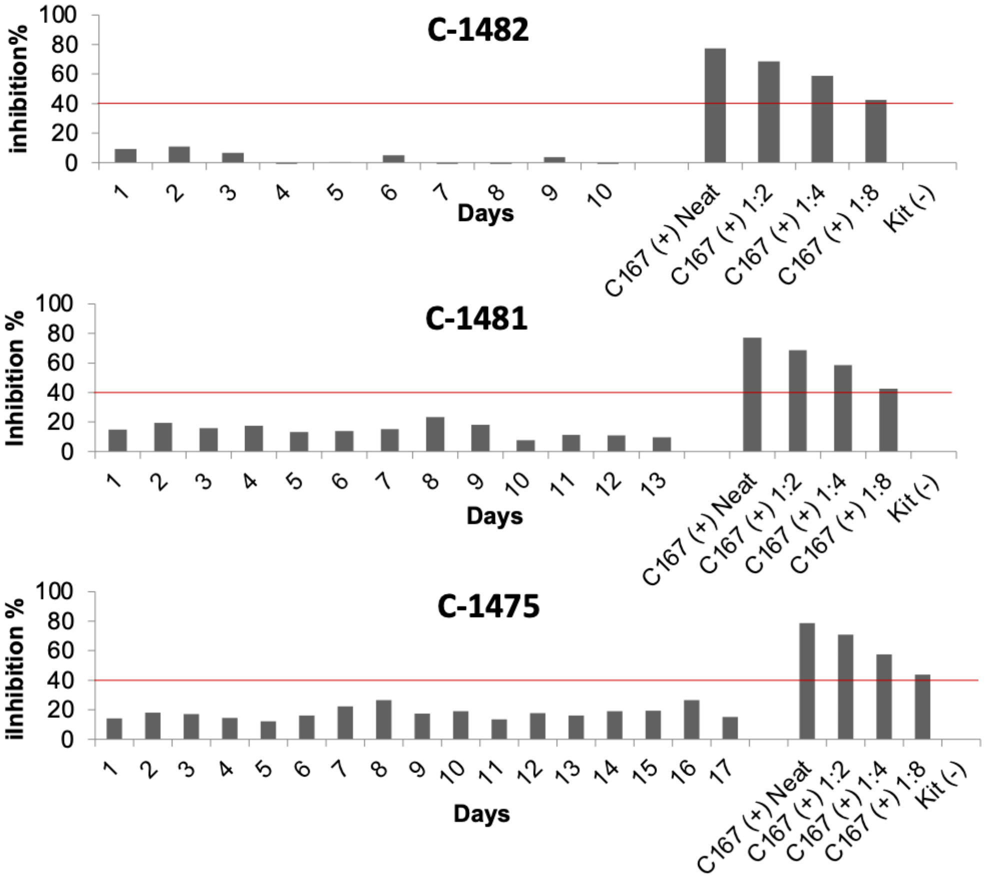

Figure S3. Analysis of Animals' serum samples from transmission experiment analysis using cELISA. Y axis represent the inhibition percent and the X axis represents the number of post-inoculation that the animal serum was collected on. In addition to the positive and negative controls are presented. Red line represents the cutoff value. Inhibition values above the line consider positive and below the line consider negative.
